# Genes selection using deep learning and explainable artificial intelligence for chronic lymphocytic leukemia predicting the need and time to therapy

**DOI:** 10.3389/fonc.2023.1198992

**Published:** 2023-08-31

**Authors:** Fortunato Morabito, Carlo Adornetto, Paola Monti, Adriana Amaro, Francesco Reggiani, Monica Colombo, Yissel Rodriguez-Aldana, Giovanni Tripepi, Graziella D’Arrigo, Claudia Vener, Federica Torricelli, Teresa Rossi, Antonino Neri, Manlio Ferrarini, Giovanna Cutrona, Massimo Gentile, Gianluigi Greco

**Affiliations:** ^1^ Biotechnology Research Unit, ‘A. Sforza’ Foundation, Cosenza, Italy; ^2^ Department of Mathematics and Computer Science, University of Calabria, Cosenza, Italy; ^3^ Mutagenesis and Cancer Prevention Unit, Istituto di Ricovero e Cura a Carattere Scientifico (IRCCS) Ospedale Policlinico San Martino, Genoa, Italy; ^4^ Tumor Epigenetics Unit, Istituto di Ricovero e Cura a Carattere Scientifico (IRCCS) Ospedale Policlinico San Martino, Genoa, Italy; ^5^ Molecular Pathology Unit, Istituto di Ricovero e Cura a Carattere Scientifico (IRCCS) Ospedale Policlinico San Martino, Genoa, Italy; ^6^ Consiglio Nazionale delle Ricerche, Istituto di Fisiologia Clinica del Consiglio Nazionale delle Ricerche (CNR), Reggio Calabria, Italy; ^7^ Department of Oncology and Hemato-Oncology, University of Milan, Milan, Italy; ^8^ Laboratory of Translational Research, Azienda Unità Sanitaria Locale - Istituto di Ricovero e Cura a Crabtree Scientifico (USL-IRCCS) of Reggio Emilia, Reggio Emilia, Italy; ^9^ Scientific Directorate, Azienda Unità Sanitaria Locale - Istituto di Ricovero e Cura a Carattere Scientifico (USL-IRCCS) of Reggio Emilia, Reggio Emilia, Italy; ^10^ Unità Operariva (UO) Molecular Pathology, Ospedale Policlinico San Martino Istituto di Ricovero e Cura a Carattere Scientifico (IRCCS), Genoa, Italy; ^11^ Hematology Unit, Department of Onco-Hematology, Azienda Ospedaliera (A.O.) of Cosenza, Cosenza, Italy; ^12^ Department of Pharmacy and Health and Nutritional Sciences, University of Calabria, Cosenza, Italy

**Keywords:** chronic lymphocytic leukemia, gene expression profile, deep learning, explainable artificial intelligence, feature selection

## Abstract

Analyzing gene expression profiles (GEP) through artificial intelligence provides meaningful insight into cancer disease. This study introduces DeepSHAP Autoencoder Filter for Genes Selection (DSAF-GS), a novel deep learning and explainable artificial intelligence-based approach for feature selection in genomics-scale data. DSAF-GS exploits the autoencoder’s reconstruction capabilities without changing the original feature space, enhancing the interpretation of the results. Explainable artificial intelligence is then used to select the informative genes for chronic lymphocytic leukemia prognosis of 217 cases from a GEP database comprising roughly 20,000 genes. The model for prognosis prediction achieved an accuracy of 86.4%, a sensitivity of 85.0%, and a specificity of 87.5%. According to the proposed approach, predictions were strongly influenced by CEACAM19 and PIGP, moderately influenced by MKL1 and GNE, and poorly influenced by other genes. The 10 most influential genes were selected for further analysis. Among them, FADD, FIBP, FIBP, GNE, IGF1R, MKL1, PIGP, and SLC39A6 were identified in the Reactome pathway database as involved in signal transduction, transcription, protein metabolism, immune system, cell cycle, and apoptosis. Moreover, according to the network model of the 3D protein-protein interaction (PPI) explored using the NetworkAnalyst tool, FADD, FIBP, IGF1R, QTRT1, GNE, SLC39A6, and MKL1 appear coupled into a complex network. Finally, all 10 selected genes showed a predictive power on time to first treatment (TTFT) in univariate analyses on a basic prognostic model including IGHV mutational status, del(11q) and del(17p), NOTCH1 mutations, β2-microglobulin, Rai stage, and B-lymphocytosis known to predict TTFT in CLL. However, only IGF1R [hazard ratio (HR) 1.41, 95% CI 1.08-1.84, P=0.013), COL28A1 (HR 0.32, 95% CI 0.10-0.97, P=0.045), and QTRT1 (HR 7.73, 95% CI 2.48-24.04, P<0.001) genes were significantly associated with TTFT in multivariable analyses when combined with the prognostic factors of the basic model, ultimately increasing the Harrell’s c-index and the explained variation to 78.6% (versus 76.5% of the basic prognostic model) and 52.6% (versus 42.2% of the basic prognostic model), respectively. Also, the goodness of model fit was enhanced (χ2 = 20.1, P=0.002), indicating its improved performance above the basic prognostic model. In conclusion, DSAF-GS identified a group of significant genes for CLL prognosis, suggesting future directions for bio-molecular research.

## 1 Introduction

A precise prognostic methodology in chronic lymphocytic leukemia (CLL) patients is critical from the clinical standpoint since progression to a more advanced disease stage requires therapy and often implies an adverse prognosis. At first presentation/diagnosis, over three-quarters of CLL patients are classified as early/asymptomatic disease phase and not requiring immediate therapy (1). Although most patients have a low-risk profile as indicated by the high frequency of the immunoglobulin heavy chain variable (*IGHV*) gene mutated (*IGHV*mut) status (2) and the low del(17p) occurrence involving the *TP53* locus (3), the time to first treatment (TTFT) is rather heterogeneous, and it can be partially predicted using combinations of risk-associated markers, which include staging systems and β2-microglobulin (β2-M) (2, 4–8).

Despite the proven prognostic power of this approach, the clinical course of a number of patients does not follow the pattern predicted, possibly indicating the requirement for additional prognosticators. In this respect, gene expression profiles (GEP), that is, the measurement of the activity (the expression) of all genes of interest to depict a synthetic picture of cellular function, is exploited to increase the ability to predict the prognosis of CLL patients (9–11).

Although GEP datasets represent a valuable source of information in healthcare, being currently used for diagnosis, prognosis, and precision medicine of hematological malignancies (12), their analysis results are challenging for three main reasons. The first one is the *course of dimensionality*: genomic-scale datasets typically consist of a very large number of features (genes) and a relatively small number of samples (patients). The second problem concerns *imbalanced classes*: genomics data are often collected from multiple sources and stratified based on pathologies. In most cases, there is a significant difference between the number of instances in each class. Finally, sequencing data are typically collected from multiple sources, different laboratories, and sequencing tools. This results in *noisy datasets* which are difficult to analyze (13).

A bioinformatic analysis is necessary to fully realize the potential of these large-scale sequencing data for prognosis in hematological malignancies (14–16) and solid tumors (17). Machine learning (ML) approaches have been widely used to enhance the performance of diagnostic and predictive models for different diseases and CLL as well (14–20). Resources and guidelines for using ML in CLL have been made available (21, 22).

However, most ML prognostic models for CLL fail to consider numerous variables and do not account for non-linear interactions between them (22). This limits the accuracy of the models and the ability to make informed predictions about the disease progression. Therefore, promising tools such as deep learning (DL) methods, a subset of ML methods based on artificial neural networks (NNs), may be used to overcome the aforementioned ML limitations. DL approaches recognize hidden patterns in large-scale datasets that are typically difficult to detect with traditional statistical and ML models. Recent studies propose and evaluate new feature selection (FS) approaches on genomic-scale datasets for cancer diagnosis and prognosis (23, 24). Such FS methodologies mainly aim at selecting the most informative genes, which can characterize classes and identify groups of patients.

Although very powerful, DL models are in general not immediately interpretable, meaning that it is difficult to understand the causal relationship between the inputs and their outcomes. This is an even more severe problem in the bioinformatics domain, where it is crucial to understand, for example, in the case of genomics, how the expression of a gene can affect the progression of oncological patients. In this context, the adoption of explainable artificial intelligence (XAI) methods has started to gain momentum for interpretability purposes as well as to enhance FS (25–27).

On the other hand, a widely used approach to overcome the *course of dimensionality* problem is to perform dimensionality reduction using autoencoders (AE) (24). While this has been proven effective, the encoding is typically a non-linear projection of the variables into a lower-dimensional space, making it difficult to provide the interpretations of the proper results.

This study introduces DeepSHAP Autoencoder Filter for Genes Selection (DSAF-GS), a novel DL and XAI-based FS method for genomics-scale data analysis. Such a method uses AEs for selecting the most informative genes without any change into the original features space, hence enhancing the explainability of the results and still exploiting the representation abilities of AEs. Such selection of genes is used to design and train a prediction model for diagnosis or prognosis. Eventually, the Shapely Additive ex-Planation (SHAP) (28) XAI method is applied to interpret the model results and select the most meaningful genes for the disease.

In the present paper, the proposed XAI method has been used to identify those genes whose expression levels are relevant for predicting the need of therapy in CLL patients from a prospective cohort of newly diagnosed Binet stage A CLL (O-CLL protocol) (29, 30) who are being monitored under a watch-and-wait strategy. This innovative approach enabled meaningful insights into CLL prognosis from genomic data by locating a group of significant genes to boost the prognostic power of a basic prognostic model. We point out that while our contribution is fully positioned within the research in oncology, our XAI method has broader applicability; in fact, from the bioinformatics and computational genomics point of view (18–20), an interesting avenue of further research is to assess its efficacy as a general feature-selection method, for instance, by considering datasets for which we already have some *a priori* semantic information on the most relevant features and by using classical comparison metrics for predictive models.

## 2 Materials and methods

### 2.1 Patients

A total of 224 of 523 newly diagnosed Binet A CLL cases belonging to the observational O-CLL1 study (clinicaltrials.gov identifier NCT00917540) were prospectively enrolled from 40 Italian institutions (29, 30) and studied for GEP. All participants provided written informed consent, and the relevant institutional review boards approved the study. The inclusion and exclusion criteria have been previously detailed (29). In particular, cases could be recruited only within 12 months of diagnosis and if they were aged <70 years and were Binet stage A. The biologic review committee confirmed the diagnosis using flow cytometry analysis and GEP analyses were centralized at Prof Ferrarini’s (Istituto Studio Tumori, Genoa, Italy) and Prof Neri’s (Fondazione Ca’ Granda, IRCCS, Ospedale Maggiore, Policlinico, Milan) labs, respectively. Recruitment began in January 2007. According to the guidelines, treatment was decided uniformly for all participating centers based on documented progressive and symptomatic disease.

### 2.2 Assessment of biological markers

Cytogenetic abnormalities involving deletions at chromosomes 11q23 and 17p13 were evaluated by FISH in a purified CD19^+^ population as previously described (31). *IGHV* gene mutational status was assessed on cDNA specimens (32). Sequences were aligned to the IMGT directory and analyzed using IMGT/VQUEST software. *NOTCH1* mutation hotspot was set by next-generation deep sequencing as previously described (29).

### 2.3 GEP analysis

GEP experiments were performed as previously described (29, 30). Briefly, total RNA fraction was obtained from CD19^+^-enriched B-cell samples (EasySep-Human B cell enrichment kit without CD43 depletion, Stem Cell Technologies, Voden Medical Instruments S.p.A, Milan, Italy) using the fully automated protocol of immunomagnetic cell separation with RoboSepTM (Stem Cell Technologies). Purified B-cells (CD19+) exceeded 95% were employed as total RNA sources for GEP analysis.

Preparation of DNA single-stranded sense target, hybridization to GeneChip^®^ Gene 1.0 ST Array (Affymetrix, Santa Clara, CA), and scanning of the chips (7G Scanner, Affymetrix) were carried out according to manufacturer’s protocols. RNA fraction was obtained from samples using Trizol reagent (Life Technologies, Monza, Italy). RNA quality was assessed using the Agilent 2100 Bioanalyzer (Agilent Technologies). The raw intensity expression values were processed by robust multi-array average (RMA) procedure 19 with the reannotated Chip definition files (CDF) from BrainArray libraries version 15.0.0 20 available at http://brainarray.mbni.med.umich.edu, as previously described (22). The gene and miRNA expression data have been deposited at the National Center for Biotechnology Information (NCBI) Gene Expression Omnibus repository (http://www.ncbi.nlm.nih.gov/geo/) and are accessible through GEO Series accession number GSE40570.

### 2.4 O-CLL dataset

For each patient, 19,367 genes profiles were provided. Patients are labeled according to the occurrence of an event (or not). The considered outcome was the need for therapy starting or death [dichotomous, not (event=0) *vs* yes (event=1)]. From the 217 patients in the final dataset, 120 were labeled as event=0 and 97 as event=1.

### 2.5 Feature selection

GEP studies generate large, high-dimensional, and unbalanced datasets, where each sample can have up to thousands of variables. This results in high computational costs and the possibility of overfitting. Such overfitting may mistake small changes in the data as significant differences, leading to misclassification errors. This study addresses these risks by applying FS techniques to reduce the dimensionality problem by selecting the most relevant features and removing noise and redundancy. FS techniques can be filter-based, wrapper, or embedded (33–35). The integration of multiple FS techniques is denoted as hybrid FS.

The proposed DSAF-GS approach is a hybrid FS method that combines filter-based and wrapper techniques to achieve a representative and meaningful subset of genes. DSAF-GS uses autoencoders (AE) as wrappers along with statistical filters to remove redundant genes. An NN is trained on the remaining genes as an event predictor. Finally, the SHAP XAI method is used to evaluate the contribution of genes to NN decisions. The genes with the strongest contribution are selected.

### 2.6 Neural networks

NNs are ML computational models inspired by the structure and function of the human brain. They consist of consecutive layers of interconnected artificial neurons, which process and transmit information through weighted connections. Training an NN amounts to providing a dataset of input-output pairs and identifying, *via* proper optimization methods, the NN parameters that minimize some given loss function, usually meant to measure the distance between the output at hand and the result of the NN computation on the given input. In a research context, NNs can be trained on large datasets of genetic data to identify patterns and predict the effects of genetic mutations on traits of interest (36–38). These predictions can then be used to understand further the genetic basis of diseases (38) and other phenotypic traits (39, 40) and inform the development of personalized medical treatments (37, 41). In addition, by using NNs and other computational and experimental methods (e.g. clustering and statistical analysis, such as F-test or XAI) researchers can gain deeper insights into the complex interactions between genetics and biology.

Autoencoders (AE) are particular architectures of NNs that uncover the underlying structure of the data and generate a latent code for further analysis (42). An AE maps an input to a lower dimensional representation (latent code). Such code is expected to have uncorrelated features, being able to reconstruct the original input data. Therefore, AEs can be used for dimensionality reduction, denoising, and data generation.

### 2.7 SHapley additive exPlanation

The black-box nature of NNs often limits the interpretability of their results. Advances in XAI provide various methods for interpreting black-box models, offering a clearer understanding of their predictions. For example, Shapely Additive ex-Planation (SHAP) is a game theory-based approach for interpreting black-box models. SHAP determines the importance of a feature by observing the variations in predictions when the feature is included or excluded from the model. It assigns an importance value, called a SHAP value, to each feature, based on its contribution to the predictions (28). SHAP values are quantitative estimates, indicating ‘how’ and ‘how much’ every single gene contributes to the model decisions, providing insight into the gene’s role in the event prediction. The SHAP method provides a way to understand the underlying workings of NNs predictions, leading to improved insights and better decision-making.

An alternative XAI approach, named LIME (Local Interpretable Model-Agnostic Explanations) (43), approximates the behavior of complex models *via* local interpretable explanations. Such explanations are obtained by fitting simpler and interpretable surrogate models with perturbed input data and observing the resulting changes in the model’s predictions.

While LIME approximates the behavior of complex models with simpler ones, SHAP provides a more direct and explicit connection between feature importance and predictions. This transparency, along with a solid theoretical foundation rooted in game theory, enhances a deeper understanding of the underlying mechanisms driving the model’s decisions.

### 2.8 Proposed algorithm

**Figure 1:**
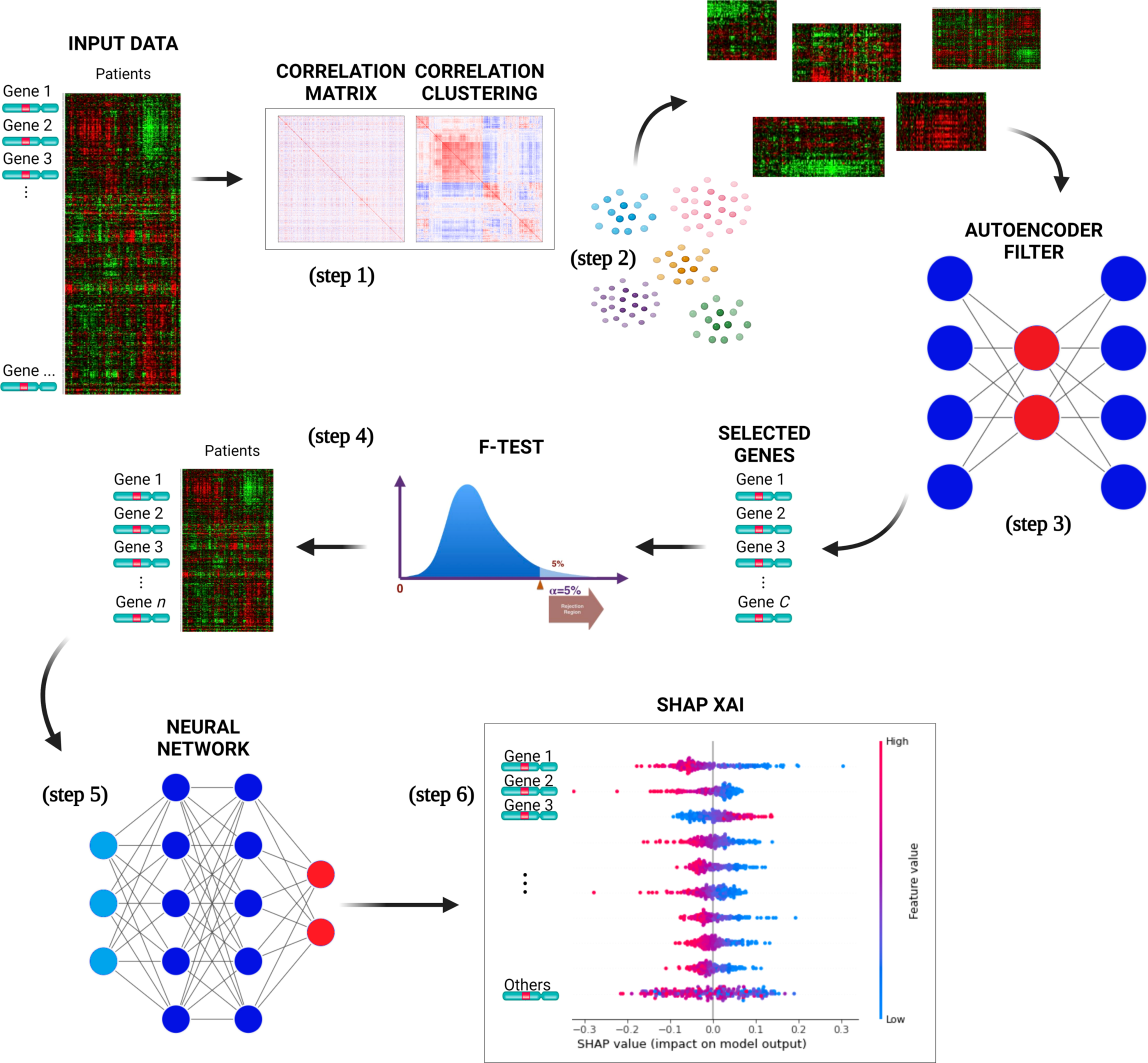
The pipeline proposed for selecting a subset of genes relevant to predict CLL events. The input data is used to compute the genes pairwise correlation matrix (step 1), and the correlation matrix is clustered (step 2) to group similarly correlated genes. The clusters are then mapped to the original input data and transposed. AEs are trained for each cluster to select the most representative gene, reducing dimensionality (step 3). The genes are ranked with F-test, selecting a subset with the highest F-value (step 4). A neural network is trained with a selected set of genes to perform binary classification of the CLL patients (event=0 and event=1) (step 5). The best NNs architecture is determined through model selection, and the SHAP XAI method explains each gene’s importance in the predictions (step 6).

DSAF-GS consists of the following steps (**Figure 1**):

1. The pairwise correlation (Pearson) was computed over the whole set of genes.
2. The resulting correlation matrix was clustered using hierarchical clustering such that similarly correlated genes belong to the same cluster.
3. For each cluster, all patient data were retrieved from the original dataset. An AE is then trained for each cluster using patients as features and genes as samples. The AEs selected the most representative gene of the respective cluster. The most representative gene was the one associated with the lowest reconstruction error. This step eliminates redundant genes hence reducing the dimensionality, still working at the level of the original feature.
4. Genes selected in the previous step were then ranked with the F-test. The F-value was computed by considering, for each gene, the ratio of the variance between and within the groups (event=0 and event=1). A subset of genes with the highest F-value was then selected. The final subset size was empirically selected by considering different subset sizes.
5. A NN was trained to perform binary classification on the event class, using the previously selected set of genes as input variables and by considering the standard binary cross-entropy loss function. A model selection phase identified the most appropriate architecture of the NN. A grid-search approach is applied over a hyperparameters space defined by the number of layers and neurons per layer. In particular, we considered 2, 3, and 4 layers and 8, 16, 32, 64, 128, and 256 neurons for the first layer. In every successive layer, the number of neurons was determined as half of the number of neurons in the preceding layer. For each given configuration (layers/neurons), we built the corresponding NN whose performances were evaluated using a cross-validation (cv) algorithm, which assesses the model’s ability to generalize by repeatedly training and testing on different subsets of the data for multiple iterations. The hyperparameters configuration is chosen as the one with the highest average performances (according to the average binary accuracy) over the cv iterations; the best model is finally selected as the one with the best performances among the cv iterations of the chosen configuration.
6. SHAP XAI method was used to explain the chosen NN classifier for the CLL event. SHAP evaluates the importance of each gene on the predictions by providing information on how such genes affect the prognosis.

### 2.9 Implementation

The DSAF-GS algorithm has been implemented using the Python (v3.8.11) programming language. NNs have been implemented using the Tensorflow (v2.6.0) framework and the Keras library. XAI analysis was performed using the SHAP (28) library.

For GEP analysis, 500 clusters were identified (step 2). Each AE architecture (step 3) consisted of five layers with the following number of neurons: 217, 43, 21, 43, and 217, respectively; *relu* was used as activation function and *Adam* as optimizer with a learning rate of 0.01; *Mean Absolute Error* was used as reconstruction loss, and each AE was trained for 1000 epochs. Out of the 500 genes selected by the AEs (one for each cluster), different subset sizes were used to train the NN event predictor. The F-test (step 4) was used to select a subset of genes of size 5, 10, 50, 100, and 300 (**Table 1**). Different NNs were trained (step 5) for each gene subset. The best model for GEP takes in 50 genes as input and consists of 4 layers of 46, 22, 12, and 1 neuron, respectively.

**Table 1:** Models’ accuracy in the binary classification of CLL event (i.e., therapy need or death).

Number of genes	Best accuracy (%)	95% Confidence Interval
**5**	79.54	70.88-77.74
**10**	84.09	72.60-78.30
**50**	86.36	65.60-75.30
**100**	79.54	65.82-72.36
**300**	75.00	66.36-70.90

During grid-search (step 5), *Adam* was used as an optimizer with a learning rate of 0.001, and *relu* was used as an activation function. A total of 10 cv iterations have been performed for each configuration by randomly splitting the data into a training set and test set (90% and 10% of the whole dataset, respectively). While the training set has been enriched with synthetic samples (by using SMOTE) (44) to guarantee a balanced training of the NN, the test set only comprised real data samples. While the computation of SHAP values was found to be inefficient for NN models, the authors (36) demonstrated that Shapley values could be calculated through a weighted linear least square regression with a shapely kernel. Such a method was adopted for computing SHAP values (step 6) using a subsample of 100 patients. Note training and test sets are different parts of the same O-CLL dataset. Indeed, we are not aware of any further public dataset having the clinical and genomic information required by our method and that can be used as a validation set, by fitting our needs and the prospective nature of our study.

### 2.10 Statistical analysis

**Figure 2:**
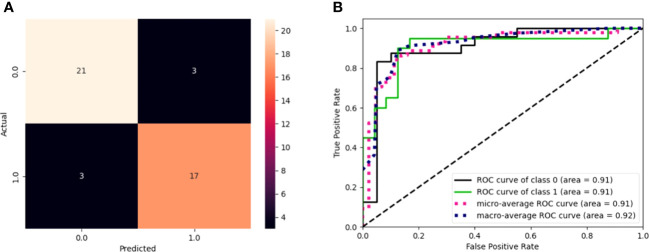
**(A)** Confusion matrix of model performance on the test set in predicting the event or non-event of new patients. Black squares refer to wrongly classified patients (false positives and false negatives), while colored squares refer to well-classified patients (true positives and true negatives). **(B)** ROC curves for the model. The graph plot sensitivity against specificity at various threshold settings. The classifier performs better as the curve approaches the upper left corner. An AUC value of 0.91 for GEP predictions indicates the solid overall performance of the model.

TTFT was calculated during the watch and wait period from the date of the diagnosis to the date of therapy start or last follow-up. The prognostic impact of predictors was investigated by univariable and multiple Cox regression analysis. Data were expressed as hazard ratio (HR) and 95% confidence intervals (CIs). The predictive accuracy of the prognostic models was quantified by calculating Harrell’s c-index (HC-index), ranging from 0.5 to 1.0, and the explained variation on the outcome (i.e., an index combining calibration and discrimination) (45). The improvement of model fitting due to the inclusion of specific genes was assessed by the log-likelihood ratio statistics. The receiver operator curves (ROC) **Figure 2B** illustrate the model performance by plotting the actual positive rate (sensitivity) versus the false positive rate (1 - specificity). A value of P <.05 was considered significant for all statistical calculations. Data analysis was performed by SPSS for Windows v.21, IBM, Chicago, Illinois, USA, and by Stata 16, StataCorp, Texas, USA.

### 2.11 Pathway and gene network analysis

Pathway analysis was performed using the Reactome Pathway Analysis tool (reactome.org) to group genes into specific pathways. Reactome analysis with statistical hypergeometric distribution test determines whether certain pathways are over-represented in the submitted data. This test produces a probability score, which is corrected for false discovery rate using the Benjamani-Hochberg method (46, 47).

Gene network was constructed with the free NetworkAnalyst tool (48) using IMEx Interactome [Literature-curated comprehensive data from InnateDB (49)].

## 3 Results

### 3.1 Gene selection by explainable artificial intelligence

Of the 224 enrolled patients, 217 were used for the analysis. The remaining 7 were removed for defective gene profiles. The performance of the best model on the test set is reported in **Table 1**. The results are shown according to the number of genes used as independent variables (first column). The best model accuracy is 86.36%, achieved using 50 genes as predictors, with a sensitivity and specificity of 85% and 87.5%, respectively. The second column reports the 95% CIs computed using the model accuracy’s mean and standard deviation over the cv iterations.

The model sensitivity and specificity are reported in **Figures 2A, B**, in terms of a confusion matrix and ROC curve, respectively. Despite the three false positives and three false negatives, the model is capable of detecting the underlying patterns in the data, as shown by the overall performance. **Figure 2B** shows that the models have an area under the curve (AUC) of 0.91.

**Figure 3:**
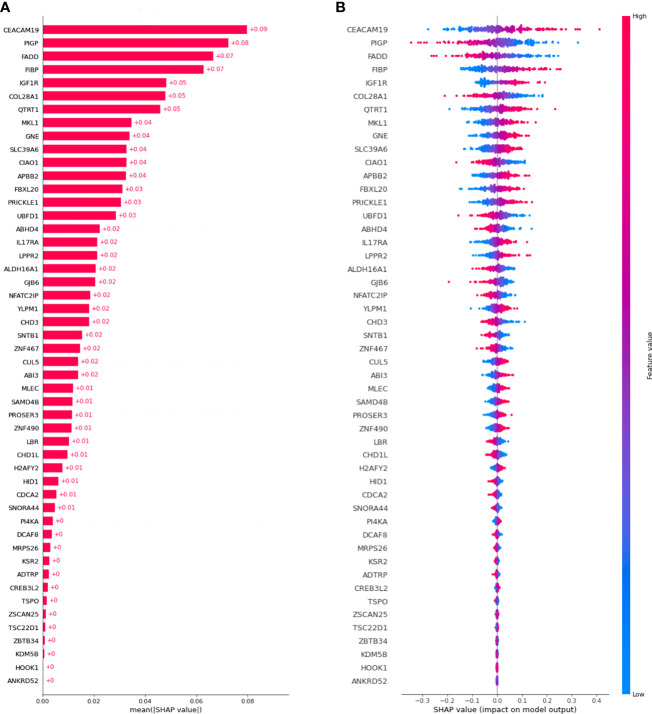
SHAP values were computed for the best model. **(A)** Waterfall plot of absolute mean SHAP values (average absolute importance of each gene in the model), **(B)** Beeswarm plot of SHAP values (shows how and how much each gene influences the predictions).

**Figure 3A** shows a waterfall plot of absolute mean SHAP values, reporting the average importance of each gene in the model, evaluated using SHAP. Genes are reported in order of importance. For example, the model was strongly influenced by *CEACAM19* and *PIGP* (+0.09 and +0.08), moderately influenced by *MKL1 (alias of MRTFA gene)* and *GNE* (+0.04), and poorly influenced by others (<0.03).

Moreover, as shown in **Figure 3B**, higher values of the gene *CEACAM19* are associated with positive SHAP values, meaning that they will increase the prediction towards the occurrence of the event. Moreover, lower values of the variable are associated with negative SHAP values, meaning that they will decrease the prediction towards the absence of an event. Conversely, for the *PIGP* gene lower values are associated with positive SHAP values, increasing the prediction towards the event’s occurrence. *PIGP* higher values are associated with negative SHAP values, meaning they will decrease the forecast towards no event occurrence.

### 3.2 Pathways and networks overview based on Reactome database of the top 10 genes

**Figure 4:**
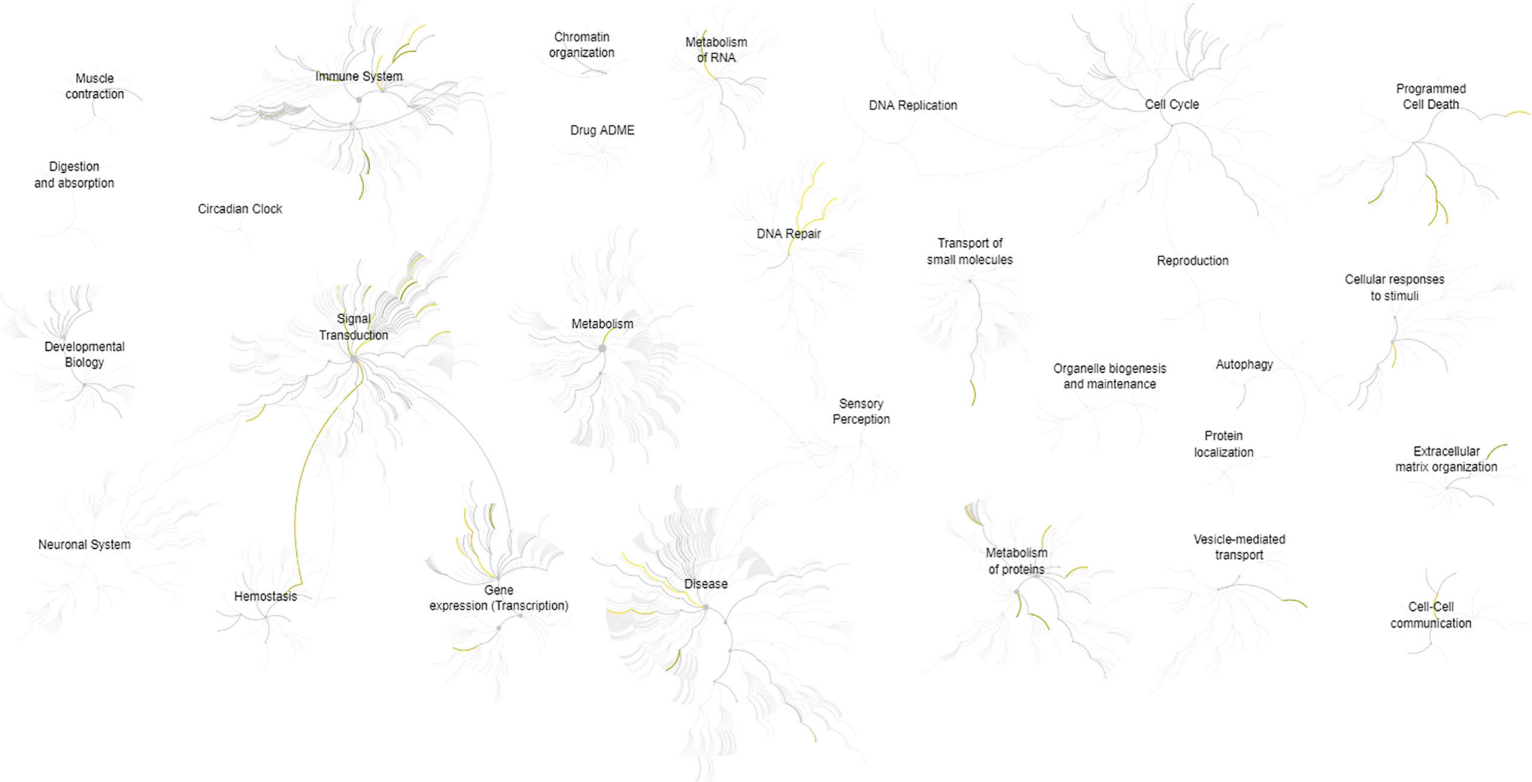
Pathways overview based on the Reactome database of the top 10 genes identified by the (SHAP) XAI method. A genome-wide overview of the results of pathway analysis is shown. Reactome pathways are arranged in a hierarchy. The center of each of the circular “bursts” is the root of one top-level pathway, for example, Cell Cycle. Each step away from the center represents the next lower level in the pathway hierarchy. The color code denotes the over-representation of that pathway in the input dataset. The closer the color is to yellow, the more significant the over-represented pathway is; light grey indicates pathways that are not significantly over-represented.

The description and chromosome localization of the top ten genes are described in **Table 2**. Eight of the top ten genes (i.e., *FADD, FIBP, FIBP, GNE, IGF1R, MKL1, PIGP, SLC39A6*) selected by the NN algorithm were found in the Reactome pathway database, showing involvement in various pathways such as signal transduction, gene expression (transcription), protein metabolism, immune system, cell cycle and apoptosis (**Figure 4**).

**Table 2:** Description and localization of the top ten genes derived from SHAP analysis.

Gene name	Chromosome	Gene start (bp)	Gene end (bp)	Gene description
**CEACAM19**	19	44662278	44684359	CEA cell adhesion molecule 19 [Source:HGNC Symbol;Acc:HGNC:31951]
**PIGP**	21	37062846	37073170	Phosphatidylinositol glycan anchor biosynthesis class P [Source:HGNC Symbol;Acc:HGNC:3046]
**FADD**	11	70203296	70207390	Fas associated *via* death domain [Source:HGNC Symbol;Acc:HGNC:3573]
**FIBP**	11	65883740	65888531	FGF1 intracellular binding protein [Source:HGNC Symbol;Acc:HGNC:3705]
**IGF1R**	15	98648539	98964530	insulin like growth factor 1 receptor [Source:HGNC Symbol;Acc:HGNC:5465]
**COL28A1**	7	7356203	7535873	Collagen type XXVIII alpha 1 chain [Source:HGNC Symbol;Acc:HGNC:22442]
**QTRT1**	19	10701430	10713437	Queuine tRNA-ribosyltransferase catalytic subunit 1 [Source:HGNC Symbol;Acc:HGNC:23797]
**MKL1 (MRTFA)**	22	40410281	40636719	Myocardin related transcription factor A [Source:HGNC Symbol;Acc:HGNC:14334]
**GNE**	9	36214441	36277042	Glucosamine (UDP-N-acetyl)-2-epimerase/N-acetylmannosamine kinase [Source:HGNC Symbol;Acc:HGNC:23657]
**SLC39A6**	18	36108531	36129385	Solute carrier family 39 member 6 [Source:HGNC Symbol;Acc:HGNC:18607]

**Figure 5:**
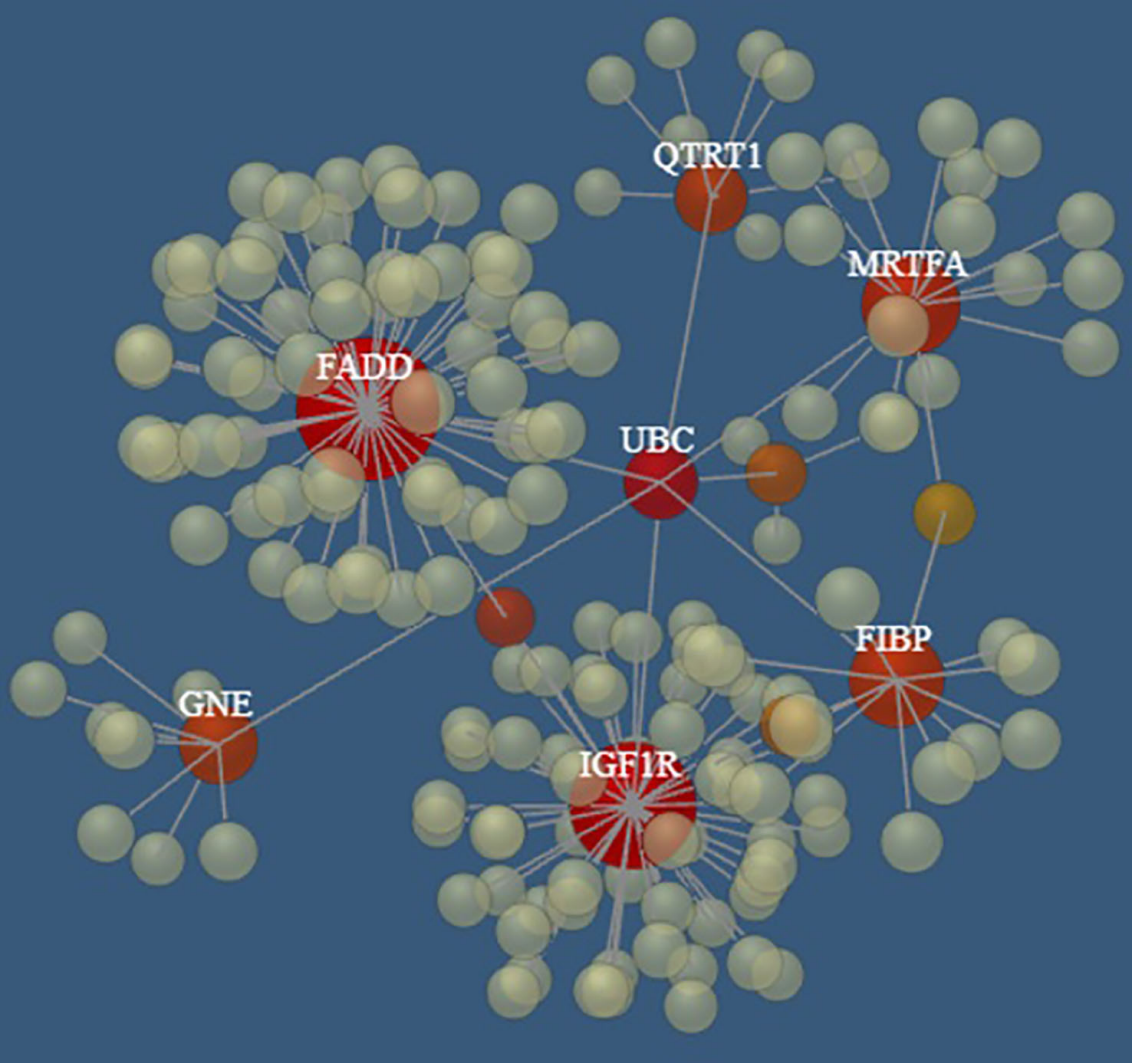
The PPI networks created by *FADD, FIBP, IGF1R, QTRT1, GNE, SLC39A6*, and *MRTFA* genes. Node size and color correspond to the number of connected edges; gene name is displayed only for nodes with ≥ 4 edges, and the closer the color is to red, the bigger the node size is.

Interestingly, seven (i.e., *FADD*, *FIBP, IGF1R, QTRT1, GNE, SLC39A6, MKL1*) of the top 10 genes appear to be connected into a complex network as shown in **Figure 5** by the network model of the 3D protein-protein interaction (PPI) explored using the NetworkAnalyst tool (48).

### 3.3 Multivariate analysis of the top 10 genes

The top 10 genes selected by the NN models were chosen to estimate their prognostic influence on noticeable clinical and biomolecular variables (named basic prognostic model) consisting of *IGHV* mutational status, del(11q) and del(17p), NOTCH1 mutation, β2-M, Rai stage, and B-lymphocytosis. **Table 3** shows their prediction power on TTFT in univariable analysis.

**Table 3:** Univariable Cox analyses for time to first treatment of several well-known clinical and biomolecular variables belonging to the basic prognostic model.

	HR	LL 95%CIs	UL 95%CIs	P-value
***IGHV* unmutated**	5.35	3.95	7.26	<.001
**del(11q)**	5.31	3.51	8.04	<.001
**del(17p)**	5.20	2.42	11.17	<.001
**NOTCH1 mutated**	2.51	1.74	3.61	<.001
**β2-M abnormal (level/cutoff…)**	2.21	1.56	3.14	<.001
**Rai stage I-II**	1.92	1.39	2.65	<.001
**B-Lymphocytes (≥5×10^9^/L)**	2.15	1.48	3.12	<.001

β2-M, β2-microglobulina; IGHV, immunoglobulin heavy chain gene rearrangement; LL, lower limit; UL, upper limit; Cis, confidence intervals.

**Figure 6:**
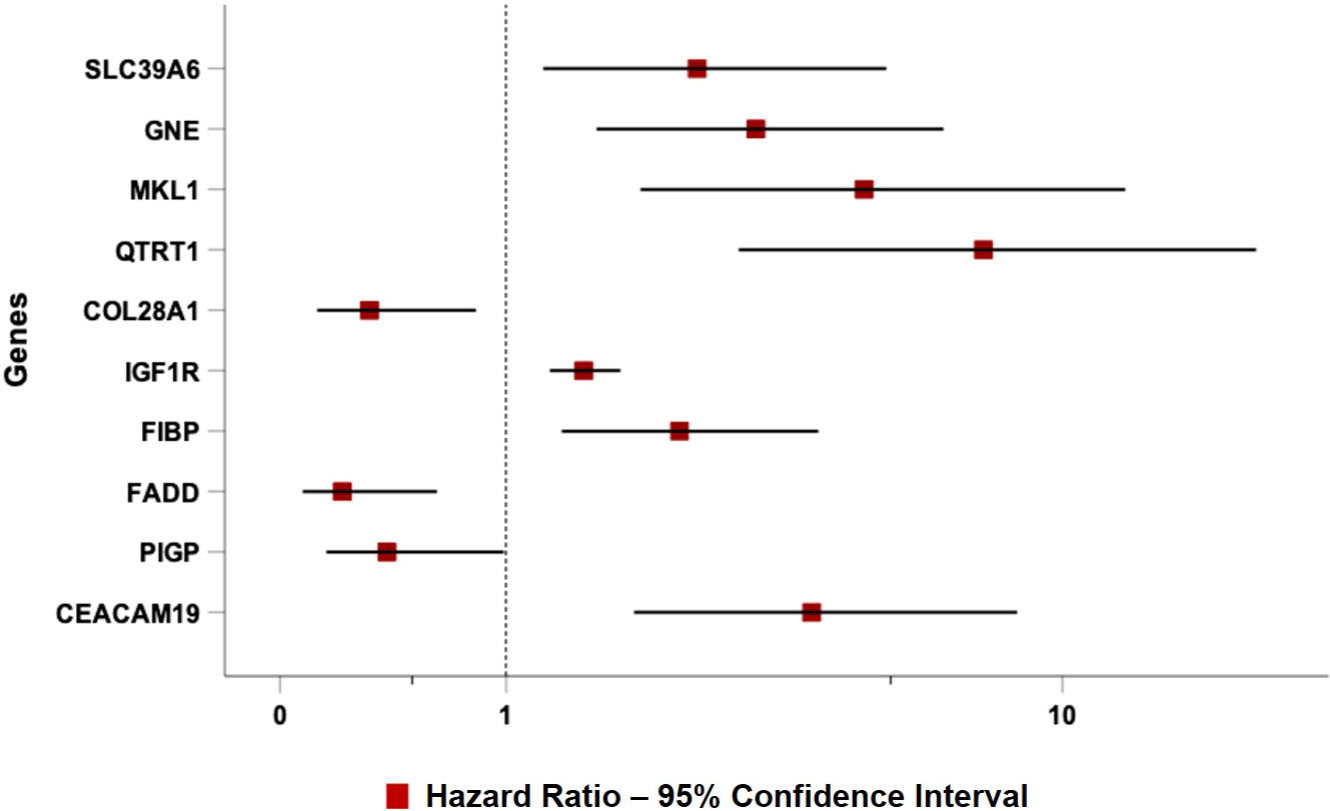
Forest plot of Cox univariable analysis for time to TTFT according to the top 10 genes selected by the NN algorithm.

As expected, all ten top genes showed a predictive power on TTFT in univariable analyses (**Figure 6**). Specifically, for *COL28A1*(HR 0.32, 95% CI 0.12-0.82, P=0.018), *FADD* (HR 0.21, 95% CI 0.07-0.62, P=0.005), and *PIGP* (HR 0.39, 95% CI 0.15-0.98, P=0.047) high expression was associated with a reduced risk to be treated, while the remaining genes showed an inverse prognostic association with therapy need. However, *CEACAM19* (HR 2.44, 95% CI 0.84-7.14, P=0.10), *PIGP* (HR 0.57, 95% CI 0.19-1.72, P=0.32), *FADD* (HR 0.45, 95% CI 0.12-1.64, P=0.22), *FIBP* (HR 1.95, 95% CI 0.96-3.96, P=0.06), *MKL1* (HR 2.31, 95% CI 0.73-7.34, P=0.15), *GNE* (HR 1.86, 95% CI 0.82-4.26, P=0.14) and *SLC39A6* (HR 1.44, 95% CI 0.64-3.22, P=0.37) lost their independent predictive power when analyzed with variables belonging to the basic prognostic model. Conversely, *IGF1R* (HR 1.41, 95% CI 1.08-1.84, P=0.013), *COL28A1* (HR 0.32, 95% CI 0.10-0.97, P=0.045), and *QTRT1* (HR 7.73, 95% CI 2.48-24.04, P<0.001) genes were significantly associated with TTFT in multivariable analyses (**Table 4**). When these three significant genes were evaluated in a final multivariable model, including the basic prognostic variables, *COL28A1*, along with B-lymphocytosis, lost its significance, while *IGF1R* and *QTRT1* maintained their prognostic independence (**Table 4**).

**Table 4:** Cox multivariable analyses for time to first treatment (TTFT).

Model 1	HR	LL 95% CI	UL 95% CI	P-value
***IGHV* unmutated**	2.03	1.12	3.70	0.02
**del(11q)**	3.92	1.77	8.69	<.001
**del(17p)**	11.86	2.51	56.02	0.002
**NOTCH1 mutated**	2.07	1.11	3.86	0.021
**β2-M abnormal**	2.10	1.33	3.31	0.001
**Rai stage I-II**	1.63	1.00	2.68	0.05
**B-Lymphocytes ≥5×10^9^/L**	1.75	0.88	3.48	0.112
**IGF1R gene**	1.41	1.08	1.84	0.013
Model 2				
***IGHV* unmutated**	2.78	1.58	4.89	<.001
**del(11q)**	2.88	1.34	6.20	0.007
**del(17p)**	7.28	1.60	33.10	0.01
**NOTCH1 mutated**	2.26	1.20	4.22	0.011
**β2-M abnormal**	2.04	1.300	3.20	0.002
**Rai stage I-II**	1.70	1.04	2.78	0.035
**B-Lymphocytes ≥5×10^9^/L**	1.42	0.72	2.80	0.313
**COL28A1 gene**	0.32	0.10	0.97	0.045
Model 3				
***IGHV* unmutated**	2.465	1.40	4.34	0.002
**del(11q)**	2.42	1.12	5.21	0.024
**del(17p)**	6.78	1.48	31.15	0.014
**NOTCH1 mutated**	2.22	1.19	4.13	0.012
**β2-M abnormal**	1.79	1.13	2.85	0.013
**Rai stage I-II**	1.93	1.17	3.18	0.01
**B-Lymphocytes ≥5×10^9^/L**	1.60	0.81	3.17	0.177
**QTRT1 gene**	7.73	2.48	24.04	<.001
Final Model				
***IGHV* unmutated**	1.93	1.06	3.50	0.031
**del(11q)**	3.10	1.39	6.92	0.006
**del(17p)**	8.44	1.75	40.75	0.008
**NOTCH1 mutated**	2.21	1.17	4.14	0.014
**β2-M abnormal**	1.97	1.23	3.15	0.005
**Rai stage I-II**	1.71	1.03	2.83	0.038
**B-Lymphocytes ≥5×10^9^/L**	1.85	0.91	3.74	0.088
**IGF1R**	1.38	1.07	1.79	0.014
**COL28A1**	0.52	0.16	1.67	0.273
**QTRT1**	6.70	2.12	21.21	0.001

In models 1-3, the basic prognostic model variables were included in the multiple models with every single gene. The results of the analyses in which the specific gene was independently associated with TTFT are reported. In the final model, the three significant genes were integrated into the multivariable analysis with the markers of the basic prognostic model.

β2-M, β2-microglobulin; LL, lower limit, UL, upper limit; CIs, confidence intervals.

The basic prognostic model provided an HC-index of 76.5% and an explained variation to predict the TTFT of 42.2%. When the three significant genes (i.e., *IGF1R*, *COL28A1*, and QTRT1) were jointly considered in the final multivariable model, the HC-index and the explained variation significantly increased to 78.6% and 52.6%, respectively, along with an improvement of the goodness of model fit (χ2 = 20.1, P=0.002). In a more parsimonious model, only including *IGF1R* and *QTRT1* (i.e., the two genes that remained significantly associated with the TTFT in the final model) and excluding *CLO28A1*, the HC-index (78.2%) and the explained variation (52.4%) retained a better performance as compared with the basic prognostic model, with a concomitant rise in the goodness of the model fit (χ2 = 18.8, P=0.001).

## 4 Discussion

The considerable innovations in genomics engendering a vast and miscellaneous bulk of information from sizable cohorts of patients and the concurrent computer science knowledge improvements have guided the growing use of AI and, more specifically, of ML approaches that acquire knowledge from available data, devising variable selections without pre-setting programming (50). Well-defined examples of the ML approach in the analysis of hematological malignancies are the association of BCL6 and PDL1/2 rearrangements in primary testicular diffuse large B-cell lymphoma (DLBCL) with central nervous system relapse (51); the involvement of six prognosis-related long non-coding genes in acute myeloid leukemia (AML) patients (52); or the relevance of tumor mutation burden for the DLBCL overall survival prognostication (53) are. In CLL, the ML algorithm identified six hub genes as possible biomarkers to improve the diagnosis (14). Moreover, baseline clinical data added to the international prognostic index for CLL (CLL-IPI) variables demonstrated improved predictive performance over CLL-IPI, using a range of ML boosting algorithms to identify the individual risk of death, treatment, infection, and a combination of them (16). In contrast, no additional improvement was observed when comprising recurrent genetic mutation information (16). Moreover, an ML algorithm called CLL Treatment Infection Model (CLL-TIM) was applied to recognize patients at high risk of infection and/or treatment based on CLL-IPI variables and routine clinical data (17).

However, differently from our prospective study, the CLL-IPI score system only included 32% of Binet stage A patients and, more importantly, 4% of IGHV mutated cases, thereby rendering it less representative of the real-world setting and may lower the TTFT’s predictive efficacy. In contrast, the Brno-Barcelona cohort (54) had a significantly higher proportion of early-stage/low-risk Binet A (83%) and IGHV mutated cases (43%), as did the German CLL study group which also developed a predictive model for newly diagnosed Binet stage A patients (55), with roughly 71% of the population having an IGHV mutation status.

Herein, we selected the top 10 genes (*CEACAM19, PIGP, FADD, FIBP, FIBP, GNE, IGF1R, MKL1, PIGP*, and *SLC39A6)* from a GEP dataset of 217 CLL cases comprising roughly 20,000 genes using a novel deep ML-based approach to estimate how much every single gene had a role in predicting the therapy need occurrence. The GEP model was strongly influenced by *CEACAM19* and *PIGP* (SHAP value +0.09 and +0.08) in making decisions, moderately influenced by *MKL1* and *GNE* (SHAP value +0.04), and poorly influenced by the others (SHAP value <0.03). *IGF1R*, *COL28A1*, and *QTRT1* moderately influenced quite the GEP model (SHAP value +0.05).

Some variables, namely Rai stage, *IGHV* mutational status, β2-M, and 17(p) and 11(q) deletions previously validated in the CLL-IPI score system (5, 56) and by our group (57, 58), were used as a basic risk model in predicting TTFT. We found that *IGF1R*, *COL28A1*, and *QTRT1* genes maintained their own independent prognostic value in predicting the time-to-event when tested in a multivariable model, including the variables of the basic prognostic model. However, in a final multivariable model, in which the three genes (*IGF1R*, *COL28A1*, and *QTRT1)* were tested all together with the prognostic variables of the basic model *IGF1R* and *QTRT1*, but not *COL28A*, maintained their predictive independence on TTFT. Notably, the presence of these genes in the model significantly increased the prognostic accuracy of a basic risk model. In this regard, the HC-index and the explained variation significantly increased from 76.5% in the basic model to more than 78% and from 42.2% to roughly 52% in the *IGF1R/QTRT1*-gene model, respectively. These data indicate that the *IGF1R/QTRT1*-gene model retained a better performance than the basic prognostic model.

*IGF1R* encoding the insulin-like growth factor 1 receptor (IGFR1) is not only implicated in numerous cellular bio-functional processes, i.e., growth, proliferation, differentiation, and apoptosis (59), but also it plays a critical role in cancer development, progression, and metastasis (60). Moreover, IGF1R is involved in CLL (61–63) and overexpressed in various CLL cell subsets. Its inhibition induced apoptosis and efficiently reduced CLL growth in an Eμ-TCL1 transgenic murine model (62). Moreover, IGF1R seems to be a direct target of sorafenib since the latter decreased its expression and phosphorylation by offsetting the insulin-like growth factor-1 binding to CLL cells and ultimately dropping the *in vitro* IGF1R kinase activity (62). Finally, we previously demonstrated the *IGF1R* gene expression as an independent prognostic factor related to TTFT in our O-CLL prospective cohort after a shorter follow-up (63).

Unlike the *IGF1R* gene, *QTRT1* encoding the queuine tRNA-ribosyltransferase 1, a key enzyme involved in the post-transcriptional modification of tRNAs (64), has never been implicated in the pathogenesis or prognosis of CLL. Conversely, a significant increase in *QTRT1* expression and a striking down-regulation in its methylation was also found in lung cancer (65). Furthermore, it was discovered to be a risk factor for the disease onset and progression and adversely associated with survival outcomes (65).

Among various CLL prognostic models involving genes (66–69), Herold et al. (11) provided evidence of the association between overall survival and TTFT and the expression of 8 genes in CLL cells (PS.8 score). For TTFT, PS.8 showed an improved prognostic effect than the single parameters and even to a combined FISH and *IGVH* status model, which, in turn, failed to increase the performance of the PS.8 score in a multivariable analysis.

Huang et coll (70). showed that high *NRIP1, BCL11B*, and *SIRT1* expressions were associated with more prolonged survival, while high expression of *CDKN2A* and *SREBF2* with a poor prognosis (70). However, a substantial fraction of patients in the dataset chosen by the authors was not analyzed at the diagnosis/first presentation but at the time of progressive disease or relapse (70). Conversely, patients of our O-CLL cohort were prospectively followed-up, and all the biomolecular analyses were performed at the disease onset. Moreover, both Herold’s (11) and Huang’s (70) studies did not consider, unlike our study, unavailable risk factors included in the CLL-IPI score, somewhat misinterpreting the final results.

Two recently published articles represent interesting innovations in CLL’s gene-oriented prognosis (71, 72). Liang X et al., following the super-enhancer (SE) new hypothesis, generated a prognostic score to predict the time-to-therapy-need in CLL by the expression levels of nine SE-associated genes (71). Yet, since several data suggest the high dependency of CLL cells on microenvironment support, Abrisqueta and coll (72). described the prediction power of a signature for predicting progression based on the analysis of two hundred genes linked to microenvironment signaling by the NanoString approach. This novel approach established a 15 genes-based signature that predicted disease outcome independently of the *IGHV* mutational status, the CLL-IPI, and the International Prognostic Score for Early-stage (IPS-E) CLL score (72). Notably, the nanoString platform, overcoming GEP methodological drawbacks and reproducibility, could represent the future, facilitating its use in clinical settings.

Notably, several pathways involved in cancer and hematopoietic malignancies development were identified by Reactome analysis of the top ten genes analyzed in this study, including Interferon alpha/beta signaling (73–75), caspases and Rho GTPase activity (76), *GHR* signaling pathway (77–79), Integrin signaling (80), non-receptor Tyrosine Kinases activity (81), and *FGF/FGFR* pathways (82). Moreover, among the top ten genes, *FIBP* was found to be overexpressed in a specific group of CLL patients affected by a large loss at the 13q14 locus (83); as previously noted also, *IGF1R* was identified as overexpressed in various CLL subsets, suggesting a contribution to CLL pathology (63, 81, 84, 85). Finally, seven of the top 10 genes (which appeared to be connected in a complex PPI network) are, in turn, interconnected through the *UBC* gene, encoding for Polyubiquitin C, which represents one of the sources of ubiquitin in human cells. Polyubiquitin C plays a crucial role in maintaining cellular ubiquitin levels, especially during the stress response. The process of ubiquitination has been associated with protein degradation, DNA repair, cell cycle regulation, kinase modification, endocytosis, and regulation of other cell signaling pathways (86). Interestingly, Zhang et al. (87), through bioinformatic analysis of gene expression profiles in CLL cells, identified the *UBC* gene as the key node in the PPI network of genes up-regulated in B cells co-stimulated with immobilized anti-IgM with respect to untreated cells, revealing the proteasome pathway as the most significant in this network.

Finally, it should be emphasized that this preliminary study lacks a validation cohort. To the best of our knowledge, we are not aware of any further public dataset fitting the prospective nature of our study as well as the clinical and genomic information required to answer our aims. Specifically, the GEO dataset (GSE39671) (88) does not have the characteristics required to run the method presented in this article but the characteristics are instead collected in the ICGC CLL dataset (89, 90). However, in the latter, sampling was completed within a year, as in our study, in approximately 26% of Binet A untreated CLL cases. In contrast, the median sampling time for the remaining cases of the ICGC CLL cohort was approximately 5 years (IQR 2.6-9.1), a bias that might invalidate the analysis’ conclusions. Moreover, information on the Rai stage system is lacking and the Binet stage information at sampling is not available.

## 5 Conclusions

A novel deep ML-based approach was proposed in the current analysis, exploiting the reconstruction capabilities of AEs and XAI to select the most informative genes for predicting the therapy need event. This study’s strengths lie in the use of an original ML method and the prospective nature of our study. The results, although preliminary, evidenced the effectiveness of this approach in identifying genes with independent predictive power, suggesting a set of meaningful genes for further investigation. Finally, it should be emphasized that this pilot study requires external validation using a different prospective cohort of patients with similar characteristics. Finally, it should be emphasized that this pilot study requires external validation using a different prospective cohort of patients with similar characteristics.

## Data availability statement

The original contributions presented in the study are included in the article/supplementary material. Further inquiries can be directed to the corresponding authors.

## Ethics statement

The studies involving humans were approved by Unità di Ematologia e Trapianto di Cellule Staminali, Istituto di Oncologia “Giovanni Paolo II”, Bari comitato etico Istituto di Oncologia “Giovanni Paolo II”, di Bari, Italy; Dipartimento di Ematologia, Ospedale di Venere, Bari comitato etico Ospedale di Venere, Bari, Italy; Unità Operativa Complessa Ematologia e Trapianto, Ospedale “Mons. R. Dimiccoli” - Barletta comitato etico Ospedale “Mons. R. Dimiccoli” - Barletta, Italy Divisione di Ematologia, Presidio Ospedaliero “A. Perrino”, Brindisi comitato etico Presidio Ospedaliero “A. Perrino”, Brindisi, Italy; Unità Operativa Complessa di Oncoematologia Ospedale “S. Anna e S. Sebastiano”, Caserta comitato etico Ospedale “S. Anna e S. Sebastiano”, Caserta, Italy; Divisione di Ematologia, Università di Catania Ospedale Ferrarotto, Catania comitato etico Ospedale Ferrarotto, Catania, Italy; Unità Operativa Complessa di Emato-Oncologia, Ospedale Garibaldi-Nesima, Catania comitato etico Ospedale Garibaldi - Nesima, Catania, Italy; Dipartimento di Oncologia ed Ematologia, Pugliese-Ciaccio Hospital, Catanzaro comitato etico Pugliese-Ciaccio Hospital, Catanzaro, Italy; Unità Operativa Complessa di Ematologia, Azienda Ospedaliera Cosenza comitato etico Azienda Ospedaliera Cosenza, Italy; Unità Operativa Complessa di Oncologia, Ospedale Giannettasio, Rossano Calabro, Cosenza comitato etico Ospedale Giannettasio, Rossano Calabro, Italy; Divisione di Ematologia, Ospedale Policlinico, Palermo, comitato etico Ospedale Policlinico, Palermo, Italy; Ematologia ospedale Goretti, Latina comitato etico ospedale Goretti, Latina, Italy, Clinica Ematologica, DIMI, Genova, comitato etico IRCCS Ospedale Policlinico San Martino, Genova, Italy; Oncologia medica C IRCCS Ospedale Policlinico San Martino, Genoa, Italy comitato etico IRCCS Ospedale Policlinico San Martino, Genova, Italy; Ospedale Villa Scassi Sampierdarena, Genova comitato etico Ospedale Villa Scassi, Genova, Italy; Ematologia, Azienda Ospedaliera San Martino, Genova comitato etico IRCCS Policlinico San Martino, Genova, Italy; Unità di Ematologia, Ospedale Vito Fazzi, Lecce comitato etico Ospedale Vito Fazzi, Lecce, Italy; Unità Operativa Complessa di Ematologia Ospedale di Matera comitato etico Ospedale di Matera, Italy; Divisione di Ematologia, Ospedale Papardo, Messina, Italy comitato etico Ospedale Papardo, Messina, Italy; Divisione di Ematologia, Università di Messina comitato etico Ospedale di Messina, Italy; Ematologia and Centro Trapianti Midollo Osseo, Foundation IRCCS Ca’ Granda Ospedale Maggiore Policlinico, Milano comitato etico Ospedale Maggiore Policlinico di Milano, Italy; Dipartimento di Oncologia, Ospedale Civile, Noale, Venezia comitato etico Ospedale Civile, Noale, Italy; Oncoematologia Policlinico di Modena comitato etico provinciale di Modena Italy; Divisione di Ematologia, Ospedale Cardarelli, Napoli comitato etico Ospedale Cardarelli, Napoli, Italy; Ematologia, Centro Trapianti Midollo Osseo, Azienda Ospedaliera Universitaria di Parma comitato etico Azienda Ospedaliera Universitaria di Parma, Italy; Dipartimento di Ematologia, Ospedale Santo Spirito, Pescara comitato etico Ospedale Santo Spirito, Pescara, Italy; Unità di Ematologia, Dipartimento di Onco-Ematologia, Guglielmo da Saliceto Hospital, Piacenza comitato etico Guglielmo da Saliceto Hospital, Piacenza, Italy; Unità di Ematologia, Azienda Ospedaliera of Reggio Calabria comitato etico Azienda Ospedaliera of Reggio Calabria, Italy; Unità Operativa di Ematologia, Azienda Ospedaliera Maria Nuova, Reggio Emilia comitato etico Azienda Ospedaliera Maria Nuova, Reggio Emilia, Italy; Unità Operativa Complessa di Ematologia e Trapianto di Cellule Staminali IRCCS-CROB di Rionero in Vulture, Potenza comitato etico IRCCS-CROB di Rionero in Vulture, Italy; Dipartimento di Ematologia, Ospedale Nuovo Regina Margherita, Roma comitato etico Ospedale Nuovo Regina Margherita, Roma, Italy; Ematologia, Azienda Ospedaliera Sant’Andrea, Università La Sapienza, Roma comitato etico Azienda Ospedaliera Sant’Andrea, Roma, Italy; Divisione di Ematologia, Università La Sapienza, Roma comitato etico Università La Sapienza, Roma, Italy; Unità di Ematologia e Trapianto di Cellule Staminali, IRCCS Ospedale Casa Sollievo della Sofferenza, San Giovanni Rotondo comitato etico IRCCS Ospedale Casa Sollievo della Sofferenza, San Giovanni Rotondo, Italy; Unità di Ematologia, Ospedale San Vincenzo, Taormina, comitato etico Ospedale San Vincenzo, Taormina, Italy; Unità di Ematologia, Ospedale San Nicola Pellegrino, Trani comitato etico Ospedale San Nicola Pellegrino, Trani, Italy; Centro di Riferimento Ematologico-Seconda Medicina, Azienda Ospedaliero-Universitaria, Ospedali Riuniti, Trieste comitato etico Ospedali Riuniti, Trieste, Italy; Unità Operativa Oncologia Medica, Ospedale di Circolo Fondazione Macchi, Varese comitato etico Ospedale di Circolo Fondazione Macchi, Varese, Italy; Unità Operativa di Ematologia, Ospedale dell’Angelo, Venezia-Mestre comitato etico Ospedale dell’Angelo, Venezia-Mestre, Italy; IRCCS Cà Granda-Maggiore Policlinico, Milano comitato etico Policlinico, Milano, Italy. The studies were conducted in accordance with the local legislation and institutional requirements. Written informed consent for participation was not required from the participants or the participants’ legal guardians/next of kin in accordance with the national legislation and institutional requirements.

## Author contributions

The authors confirm contributions to the paper as follows: study conception and design: FM, AN, CA, GG; data collection: CG and MG; Artificial Intelligence analysis and interpretation of results: CA, YR-A, GG; Pathways and network analysis and interpretation of results: PM, AA, FR, MC, FT, TR; Multivariate statistical analysis and interpretation of results: FM, GD’A, GT; draft manuscript preparation: CA and YR-A, and FM; results discussion and contribution to the final manuscript: AN, FM, CA, MG, CV, MF. All authors reviewed and approved the final version of the manuscript.

## References

[1] Hallek M. Chronic lymphocytic leukemia: 2020 update on diagnosis, risk stratification and treatment. Am J Hematol (2019) 94(11):1266–87. doi: 10.1002/ajh.25595 31364186

[2] Baliakas P Mattsson M Stamatopoulos K Rosenquist R. Prognostic indices in chronic lymphocytic leukaemia: where do we stand how do we proceed? J Intern Med (2016) 279(4):347–57. doi: 10.1111/joim.12455 26709197

[3] Döhner H Stilgenbauer S Benner A Leupolt E Kröber A Bullinger L. Genomic aberrations and survival in chronic lymphocytic leukemia. New Engl J Med (2000) 343(26):1910–6. doi: 10.1056/NEJM200012283432602 11136261

[4] Kreuzberger N Damen JAAG Trivella M Estcourt LJ Aldin A Umlauff L. Prognostic models for newly-diagnosed chronic lymphocytic leukaemia in adults: a systematic review and meta-analysis. Cochrane Database Systematic Rev (2020) 7(CD012022):1–233. doi: 10.1002/14651858.CD012022.pub2 PMC8078230 32735048

[5] International CLL-IPI Working Group. An international prognostic index for patients with chronic lymphocytic leukaemia (CLL-IPI): a meta-analysis of individual patient data. Lancet Oncol (2016) 17(6):779–90. doi: 10.1016/S1470-2045(16)30029-8 27185642

[6] CondoluciAdi BergamoLLangerbeinsPHoechstetterMAHerlingCDDe PaoliL. International prognostic score for asymptomatic early-stage chronic lymphocytic leukemia. Blood (2020) 135(21):1859–69. doi: 10.1182/blood.2019003453 32267500

[7] Morabito F Tripepi G Vigna E Bossio S D’Arrigo G Martino EA. Validation of the Alternative International Prognostic Score-E (AIPS-E): Analysis of Binet stage A chronic lymphocytic leukemia patients enrolled into the O-CLL1-GISL protocol. Eur J Haematol (2021) 106(6):831–5. doi: 10.1111/ejh.13614 33662164

[8] Gentile M Shanafelt TD Rossi D Laurenti L Mauro FR Molica S. Validation of the CLL-IPI and comparison with the MDACC prognostic index in newly diagnosed patients. Blood J Am Soc Hematol (2016) 128(16):2093–5. doi: 10.1182/blood-2016-07-728261 PMC5524531 27549308

[9] Rodriguez A Villuendas R Yanez L Gomez ME Diaz R Pollan M. Molecular heterogeneity in chronic lymphocytic leukemia is dependent on BCR signaling: clinical correlation. Leukemia (2007) 21(9):1984–91. doi: 10.1038/sj.leu.2404831 17611561

[10] Calin GA Ferracin M Cimmino A di Leva G Shimizu M Wojcik SE. A MicroRNA signature associated with prognosis and progression in chronic lymphocytic leukemia. New Engl J Med (2005) 353(17):1793–801. doi: 10.1056/NEJMoa050995 16251535

[11] Herold T Jurinovic V Metzeler KH Boulesteix AL Bergmann M Seiler T. An eight-gene expression signature for the prediction of survival and time to treatment in chronic lymphocytic leukemia. Leukemia (2011) 25(10):1639–45. doi: 10.1038/leu.2011.125 21625232

[12] Taylor J Xiao W Abdel-Wahab O. Diagnosis and classification of hematologic Malignancies on the basis of genetics. Blood J Am Soc Hematology. (2017) 130(4):410–23. doi: 10.1182/blood-2017-02-734541 PMC5533199 28600336

[13] Koumakis L. Deep learning models in genomics; are we there yet? Comput Struct Biotechnol J (2020) 18:1466–73. doi: 10.1016/j.csbj.2020.06.017 PMC7327302 32637044

[14] Zhu Y Gan X Qin R Lin Z. Identification of six diagnostic biomarkers for chronic lymphocytic leukemia based on machine learning algorithms. J Oncol (2022) 2022(3652107):1–19. doi: 10.1155/2022/3652107 PMC9715328 36467501

[15] Chen P El Hussein S Xing F Aminu M KannapIran A Hazle JD. Chronic lymphocytic leukemia progression diagnosis with intrinsic cellular patterns *via* unsupervised clustering. Cancers (Basel) (2022) 14(10):2398. doi: 10.3390/cancers14102398 35626003 PMC9139505

[16] Parviz M Brieghel C Agius R Niemann CU. Prediction of clinical outcome in CLL based on recurrent gene mutations, CLL-IPI variables, and (para) clinical data. Blood Adv (2022) 6(12):3716–28. doi: 10.1182/bloodadvances.2021006351 PMC9631547 35468622

[17] Agius R Brieghel C Andersen MA Pearson AT Ledergerber B Cozzi-Lepri A. Machine learning can identify newly diagnosed patients with CLL at high risk of infection. Nat Commun (2020) 11(1):363–80. doi: 10.1038/s41467-019-14225-8 PMC6969150 31953409

[18] Xie R Wen J Quitadamo A Cheng J Shi X. A deep auto-encoder model for gene expression prediction. BMC Genomics (2017) 18:39–49. doi: 10.1186/s12864-017-4226-0 29219072 PMC5773895

[19] Chen H Zhang Y Gutman I. A kernel-based clustering method for gene selection with gene expression data. J BioMed Inform (2016) 62:12–20. doi: 10.1016/j.jbi.2016.05.007 27215190

[20] Das P Roychowdhury A Das S Roychoudhury S Tripathy S. sigFeature: novel significant feature selection method for classification of gene expression data using support vector machine and t statistic. Front Genet (2020) 11:247. doi: 10.3389/fgene.2020.00247 32346383 PMC7169426

[21] Salah HT Muhsen IN Salama ME Owaidah T Hashmi SK. Machine learning applications in the diagnosis of leukemia: Current trends and future directions. Int J Lab Hematol (2019) 41(6):717–25. doi: 10.1111/ijlh.13089 31498973

[22] Agius R Parviz M Niemann CU. Artificial intelligence models in chronic lymphocytic leukemia–recommendations toward state-of-the-art. Leuk Lymphoma. (2022) 63(2):265–78. doi: 10.1080/10428194.2021.1973672 34612160

[23] Alhenawi E Al-Sayyed R Hudaib A Mirjalili S. Feature selection methods on gene expression microarray data for cancer classification: A systematic review. Comput Biol Med (2022) 140:105051. doi: 10.1016/j.compbiomed.2021.105051 34839186

[24] Danaee P Ghaeini R Hendrix DA. A deep learning approach for cancer detection and relevant gene identification. In: Pacific symposium biocomputing (2017) 2017:219–29. doi: 10.1142/9789813207813_0022 PMC5177447 27896977

[25] Graham G Csicsery N Stasiowski E Thouvenin G Mather WH Ferry M. Genome-scale transcriptional dynamics and environmental biosensing. Proc Natl Acad Sci (2020) 117(6):3301–6. doi: 10.1073/pnas.1913003117 PMC7022183 31974311

[26] Meena J Hasija Y. Application of explainable artificial intelligence in the identification of Squamous Cell Carcinoma biomarkers. Comput Biol Med (2022) 146:105505. doi: 10.1016/j.compbiomed.2022.105505 35477047

[27] Karim MR Cochez M Beyan O Decker S Lange C. OncoNetExplainer: explainable predictions of cancer types based on gene expression data, In: 2019 IEEE 19th International conference on bioinformatics and bioengineering (BIBE). Athens, Greece (2019). pp. 415–22. doi: 10.1109/BIBE.2019.00081

[28] Lundberg SM Lee SI. A unified approach to interpreting model predictions. Adv Neural Inf Process Syst (2017) 30:1–10.

[29] Morabito F Mosca L Cutrona G Agnelli L Tuana G Ferracin M. Clinical monoclonal B lymphocytosis versus Rai 0 chronic lymphocytic leukemia: a comparison of cellular, cytogenetic, molecular, and clinical features. Clin Cancer Res (2013) 19(21):5890–900. doi: 10.1158/1078-0432.CCR-13-0622 24036852

[30] Negrini M Cutrona G Bassi C Fabris S Zagatti B Colombo M. microRNAome expression in chronic lymphocytic leukemia: comparison with normal B-cell subsets and correlations with prognostic and clinical parametersmicroRNA expression in CLL. Clin Cancer Res (2014) 20(15):4141–53. doi: 10.1158/1078-0432.CCR-13-2497 24916701

[31] Fabris S Mosca L Todoerti K Cutrona G Lionetti M Intini D. Molecular and transcriptional characterization of 17p loss in B-cell chronic lymphocytic leukemia. Genes Chromosomes Cancer (2008) 47(9):781–93. doi: 10.1002/gcc.20579 18521849

[32] Fais F Ghiotto F Hashimoto S Sellars B Valetto A Allen SL. Chronic lymphocytic leukemia B cells express restricted sets of mutated and unmutated antigen receptors. J Clin Invest. (1998) 102(8):1515–25. doi: 10.1172/JCI3009 PMC509001 9788964

[33] Jović A Brkić K Bogunović N. A review of feature selection methods with applications. (2015). pp. 1200–5. doi: 10.1109/MIPRO.2015.7160458

[34] Solorio-Fernández S Carrasco-Ochoa JA Martíinez-Trinidad JF. A review of unsupervised feature selection methods. Artif Intell Rev (2020) 53(2):907–48. doi: 10.1007/s10462-019-09682-y

[35] Khaire UM Dhanalakshmi R. Stability of feature selection algorithm: A review. J King Saud University-Computer Inf Sci (2022) 34(4):1060–73. doi: 10.1016/j.jksuci.2019.06.012

[36] Daoud M Mayo M. A survey of neural network-based cancer prediction models from microarray data. Artif Intell Med (2019) 97:204–14. doi: 10.1016/j.artmed.2019.01.006 30797633

[37] Lopez-Garcia G Jerez JM Franco L Veredas FJ. Transfer learning with convolutional neural networks for cancer survival prediction using gene-expression data. PloS One (2020) 15(3):e0230536. doi: 10.1371/journal.pone.0230536 32214348 PMC7098575

[38] Flagel L Brandvain Y Schrider DR. The unreasonable effectiveness of convolutional neural networks in population genetic inference. Mol Biol Evol (2019) 36(2):220–38. doi: 10.1093/molbev/msy224 PMC6367976 30517664

[39] Ma W Qiu Z Song J Li J Cheng Q Zhai J. A deep convolutional neural network approach for predicting phenotypes from genotypes. Planta (2018) 248:1307–18. doi: 10.1007/s00425-018-2976-9 30101399

[40] Luo P Ding Y Lei X Wu FX. deepDriver: predicting cancer driver genes based on somatic mutations using deep convolutional neural networks. Front Genet (2019) 10:13. doi: 10.3389/fgene.2019.00013 30761181 PMC6361806

[41] Katzman JL Shaham U Cloninger A Bates J Jiang T Kluger Y. DeepSurv: personalized treatment recommender system using a Cox proportional hazards deep neural network. BMC Med Res Methodol (2018) 18(1):1–12. doi: 10.1186/s12874-018-0482-1 29482517 PMC5828433

[42] Bank D Koenigstein N Giryes R. Autoencoders. arXiv (2020) arXiv:2003.05991v2):1–22. doi: 10.48550/arXiv.2003.05991

[43] Ribeiro MT Singh S Guestrin C. “Why should i trust you?” Explaining the predictions of any classifier. In: Proceedings of the 22nd ACM SIGKDD International conference on knowledge discovery and data mining (KDD '16). New York, NY, USA: Association for Computing Machinery (2016). pp. 1135–44. doi: 10.1145/2939672.2939778

[44] Chawla N v Bowyer KW Hall LO Kegelmeyer WP. SMOTE: synthetic minority over-sampling technique. J Artif Intell Res (2002) 16:321–57. doi: 10.1613/jair.953

[45] Tripepi G Heinze G Jager KJ Stel VS Dekker FW Zoccali C. Risk prediction models. Nephrol Dialysis Transplantation. (2013) 28(8):1975–80. doi: 10.1093/ndt/gft095 23658248

[46] Fabregat A Sidiropoulos K Viteri G Forner O Marin-Garcia P Arnau V. Reactome pathway analysis: a high-performance in-memory approach. BMC Bioinf (2017) 18(1):1–9. doi: 10.1186/s12859-017-1559-2 PMC5333408 28249561

[47] Jassal B Matthews L Viteri G Gong C Lorente P Fabregat A. The reactome pathway knowledgebase. Nucleic Acids Res (2020) 48(D1):D498–503. doi: 10.1093/nar/gkz1031 PMC7145712 31691815

[48] Zhou G Soufan O Ewald J Hancock REW Basu N Xia J. NetworkAnalyst 3.0: a visual analytics platform for comprehensive gene expression profiling and meta-analysis. Nucleic Acids Res (2019) 47(W1):W234–41. doi: 10.1093/nar/gkz240 PMC6602507 30931480

[49] Breuer K Foroushani AK Laird MR Chen C Sribnaia A Lo R. InnateDB: systems biology of innate immunity and beyond—recent updates and continuing curation. Nucleic Acids Res (2013) 41(D1):D1228–33. doi: 10.1093/nar/gks1147 PMC3531080 23180781

[50] Li Y Wu FX Ngom A. A review on machine learning principles for multi-view biological data integration. Brief Bioinform (2018) 19(2):325–40. doi: 10.1093/bib/bbw113 28011753

[51] Twa DDW Lee DG Tan KL Slack GW Ben-Neriah S Villa D. Genomic predictors of central nervous system relapse in primary testicular diffuse large B-cell lymphoma. Blood (2021) 137(9):1256–9. doi: 10.1182/blood.2020006338 32967007

[52] Chen CT Wang PP Mo WJ Zhang YP Zhou W Deng TF. Expression profile analysis of prognostic long non-coding RNA in adult acute myeloid leukemia by weighted gene co-expression network analysis (WGCNA). J Cancer (2019) 10(19):4707. doi: 10.7150/jca.31234 31528236 PMC6746144

[53] Chen C Liu S Jiang X Huang L Chen F Wei X. Tumor mutation burden estimated by a 69-gene-panel is associated with overall survival in patients with diffuse large B-cell lymphoma. Exp Hematol Oncol (2021) 10:1–11. doi: 10.1186/s40164-021-00215-4 33722306 PMC7962318

[54] Delgado J Doubek M Baumann T Kotaskova J Molica S Mozas P. Chronic lymphocytic leukemia: a prognostic model comprising only two biomarkers (IGHV mutational status and FISH cytogenetics) separates patients with different outcome and simplifies the CLL-IPI. Am J Hematol (2017) 92(4):375–80. doi: 10.1002/ajh.24660 28120419

[55] Hoechstetter MA Busch R Eichhorst B Bühler A Winkler D Bahlo J. Prognostic model for newly diagnosed CLL patients in Binet stage A: results of the multicenter, prospective CLL1 trial of the German CLL study group. Leukemia (2020) 34(4):1038–51. doi: 10.1038/s41375-020-0727-y 32042081

[56] Gentile M Shanafelt TD Mauro FR Laurenti L Rossi D Molica S. Comparison between the CLL-IPI and the Barcelona-Brno prognostic model: analysis of 1299 newly diagnosed cases. Am J Hematol (2018) 93(2):E35–7. doi: 10.1002/ajh.24960 29098721

[57] Monti P Lionetti M De Luca G Menichini P Recchia AG Matis S. Time to first treatment and P53 dysfunction in chronic lymphocytic leukaemia: Results of the O-CLL1 study in early stage patients. Sci Rep (2020) 10(1):1–13. doi: 10.1038/s41598-020-75364-3 33116240 PMC7595214

[58] Morabito F Tripepi G Moia R Recchia AG Boggione P Mauro FR. Lymphocyte doubling time as a key prognostic factor to predict time to first treatment in early-stage chronic lymphocytic leukemia. Front Oncol (2021) 11:684621. doi: 10.3389/fonc.2021.684621 34408978 PMC8366564

[59] Werner H. For debate: the pathophysiological significance of IGF-I receptor overexpression: new insights. Pediatr Endocrinol Rev (2009) 7(1):2–5. 19696710

[60] Pollak MN Schernhammer ES Hankinson SE. Insulin-like growth factors and neoplasia. Nat Rev Cancer. (2004) 4(7):505–18. doi: 10.1038/nrc1387 15229476

[61] Schillaci R Galeano A Becu-Villalobos D Spinelli O Sapia S Bezares RF. Autocrine/paracrine involvement of insulin-like growth factor-I and its receptor in chronic lymphocytic leukaemia. Br J Haematol (2005) 130(1):58–66. doi: 10.1111/j.1365-2141.2005.05579.x 15982345

[62] Yaktapour N Übelhart R Schüler J Aumann K Dierks C Burger M. Insulin-like growth factor-1 receptor (IGF1R) as a novel target in chronic lymphocytic leukemia. Blood J Am Soc Hematology. (2013) 122(9):1621–33. doi: 10.1182/blood-2013-02-484386 23863897

[63] Maura F Mosca L Fabris S Cutrona G Matis S Lionetti M. Insulin growth factor 1 receptor expression is associated with NOTCH1 mutation, trisomy 12 and aggressive clinical course in chronic lymphocytic leukaemia. PloS One (2015) 10(3):e0118801. doi: 10.1371/journal.pone.0118801 25786252 PMC4365018

[64] Liu F Clark W Luo G Wang X Fu Y Wei J. ALKBH1-mediated tRNA demethylation regulates translation. Cell (2016) 167(3):816–28. doi: 10.1016/j.cell.2016.09.038 PMC5119773 27745969

[65] Ma Q He J. Enhanced expression of queuine tRNA-ribosyltransferase 1 (QTRT1) predicts poor prognosis in lung adenocarcinoma. Ann Transl Med (2020) 8(24):1658. doi: 10.21037/atm-20-7424 33490170 PMC7812218

[66] Mosquera Orgueira A Antelo Rodriguez B Diaz Arias JÁ Diaz Varela N Bello López JL. A three-gene expression signature identifies a cluster of patients with short survival in chronic lymphocytic leukemia. J Oncol (2019) 2019(9453539):1–4. doi: 10.1155/2019/9453539 PMC6885206 31827514

[67] Gong H Li H Yang Q Zhang G Liu H Ma Z. A ferroptosis molecular subtype-related signature for predicting prognosis and response to chemotherapy in patients with chronic lymphocytic leukemia. BioMed Res Int (2022) 2022(5646275):1–24. doi: 10.1155/2022/5646275 PMC9279058 35845961

[68] Improgo MR Tesar B Klitgaard JL Magori-Cohen R Yu L Kasar S. MYD 88 L265P mutations identify a prognostic gene expression signature and a pathway for targeted inhibition in CLL. Br J Haematol (2019) 184(6):925–36. doi: 10.1111/bjh.15714 30537114

[69] Sevov M Rosenquist R Mansouri L. RNA-based markers as prognostic factors in chronic lymphocytic leukemia. Expert Rev Hematol (2012) 5(1):69–79. doi: 10.1586/ehm.11.80 22272707

[70] Huang H y Wang Y Herold T Gale RP Wang J z Li L. A survival prediction model and nomogram based on immune-related gene expression in chronic lymphocytic leukemia cells. Front Med (Lausanne) (2022) 9:1026812. doi: 10.3389/fmed.2022.1026812 36600891 PMC9806429

[71] Liang X Meng Y Li C Liu L Wang Y Pu L. Super-Enhancer–Associated nine-gene prognostic score model for prediction of survival in chronic lymphocytic leukemia patients. Front Genet (2022) 13:1001364. doi: 10.3389/fgene.2022.1001364 36186463 PMC9521409

[72] Abrisqueta P Medina D Villacampa G Lu J Alcoceba M Carabia J. A gene expression assay based on chronic lymphocytic leukemia activation in the microenvironment to predict progression. Blood Adv (2022) 6(21):5763–73. doi: 10.1182/bloodadvances.2022007508 PMC9647836 35973197

[73] Bauvois B Pramil E Jondreville L Quiney C Nguyen-Khac F Susin SA. Activation of interferon signaling in chronic lymphocytic leukemia cells contributes to apoptosis resistance *via* a JAK-Src/STAT3/mcl-1 signaling pathway. Biomedicines (2021) 9(2):188. doi: 10.3390/biomedicines9020188 33668421 PMC7918075

[74] Bauvois B Durant L Laboureau J Barthelemy E Rouillard D Boulla G. Upregulation of CD38 gene expression in leukemic B cells by interferon types I and II. J Interferon Cytokine Res (1999) 19(9):1059–66. doi: 10.1089/107999099313299 10505750

[75] Fluckiger AC Rossi JF Bussel A Bryon P Banchereau J DeFrance T. Responsiveness of chronic lymphocytic leukemia B cells activated *via* surface Igs or CD40 to B-cell tropic factors. Blood (1992) 80(12):3173–81. doi: 10.1182/blood.V80.12.3173.3173 1281692

[76] Infante E Ridley AJ. Roles of Rho GTPases in leucocyte and leukaemia cell transendothelial migration. Philos Trans R Soc B: Biol Sci (2013) 368(1629):20130013. doi: 10.1098/rstb.2013.0013 PMC3785963 24062583

[77] Abu El-Makarem MA Kamel MF Mohamed AA Ali HA Mohamed MR Mohamed AEDM. Down-regulation of hepatic expression of GHR/STAT5/IGF-1 signaling pathway fosters development and aggressiveness of HCV-related hepatocellular carcinoma: Crosstalk with Snail-1 and type 2 transforming growth factor-beta receptor. PloS One (2022) 17(11):e0277266. doi: 10.1371/journal.pone.0277266 36374927 PMC9662744

[78] Jiang F Chen X Shen Y Shen X. Identification and validation of an m6A modification of JAK-STAT signaling pathway–related prognostic prediction model in gastric cancer. Front Genet (2022) 13:891744. doi: 10.3389/fgene.2022.891744 35928449 PMC9343854

[79] Yan HZ Wang HF Yin Y Zou J Xiao F Yi LN. GHR is involved in gastric cell growth and apoptosis *via* PI3K/AKT signalling. J Cell Mol Med (2021) 25(5):2450–8. doi: 10.1111/jcmm.16160 PMC7933969 33492754

[80] Polcik L Dannewitz Prosseda S Pozzo F Zucchetto A Gattei V Hartmann TN. Integrin signaling shaping BTK-inhibitor resistance. Cells (2022) 11(14):2235. doi: 10.3390/cells11142235 35883678 PMC9322986

[81] Siveen KS Prabhu KS Achkar IW Kuttikrishnan S Shyam S Khan AQ. Role of non receptor tyrosine kinases in hematological Malignances and its targeting by natural products. Mol Cancer (2018) 17(1):1–21. doi: 10.1186/s12943-018-0788-y 29455667 PMC5817858

[82] Sinha S Boysen J Nelson M Warner SL Bearss D Kay NE. Axl activates fibroblast growth factor receptor pathway to potentiate survival signals in B-cell chronic lymphocytic leukemia cells. Leukemia (2016) 30(6):1431–6. doi: 10.1038/leu.2015.323 PMC4879100 26598018

[83] Mosca L Fabris S Lionetti M Todoerti K Agnelli L Morabito F. Integrative genomics analyses reveal molecularly distinct subgroups of B-cell chronic lymphocytic leukemia patients with 13q14 deletionIntegrative genomics analysis of 13q14-deleted CLL. Clin Cancer Res (2010) 16(23):5641–53. doi: 10.1158/1078-0432.CCR-10-0151 20947517

[84] Kosalai ST Morsy MHA Papakonstantinou N Mansouri L Stavroyianni N Kanduri C. EZH2 upregulates the PI3K/AKT pathway through IGF1R and MYC in clinically aggressive chronic lymphocytic leukaemia. Epigenetics (2019) 14(11):1125–40. doi: 10.1080/15592294.2019.1633867 PMC6773411 31216925

[85] Scheffold A Jebaraj BMC Tausch E Bloehdorn J Ghia P Yahiaoui A. IGF1R as druggable target mediating PI3K-δ inhibitor resistance in a murine model of chronic lymphocytic leukemia. Blood J Am Soc Hematol (2019) 134(6):534–47. doi: 10.1182/blood.2018881029 PMC8212352 31010847

[86] Kliza K Husnjak K. Resolving the complexity of ubiquitin networks. Front Mol Biosci (2020) 7:21. doi: 10.3389/fmolb.2020.00021 32175328 PMC7056813

[87] Zhang L Liang A Zhang W Wang H Wang C. Bioinformatics analysis of gene expression profiles in chronic lymphocytic leukemia. Int J Clin Exp Pathol (2016) 9(9):9126–37.

[88] Chuang HY Rassenti L Salcedo M Licon K Kohlmann A Haferlach T. Subnetwork-based analysis of chronic lymphocytic leukemia identifies pathways that associate with disease progression. Blood J Am Soc Hematol (2012) 120(13):2639–49. doi: 10.1182/blood-2012-03-416461 PMC3460686 22837534

[89] Puente XS Beà S Valdés-Mas R Villamor N Gutiérrez-Abril J Martín-Subero JI. Non-coding recurrent mutations in chronic lymphocytic leukaemia. Nature (2015) 526(7574):519–24. doi: 10.1038/nature14666 26200345

[90] Knisbacher BA Lin Z Hahn CK Nadeu F Duran-Ferrer M Stevenson KE. Molecular map of chronic lymphocytic leukemia and its impact on outcome. Nat Genet (2022) 54(11):1664–74. doi: 10.1038/s41588-022-01140-w PMC10084830 35927489

